# The Long-Term Effect of Treatment Using the Transcranial Magnetic Stimulation rTMS in Patients after Incomplete Cervical or Thoracic Spinal Cord Injury

**DOI:** 10.3390/jcm10132975

**Published:** 2021-07-02

**Authors:** Agnieszka Wincek, Juliusz Huber, Katarzyna Leszczyńska, Wojciech Fortuna, Stefan Okurowski, Krzysztof Chmielak, Paweł Tabakow

**Affiliations:** 1 Department of Pathophysiology of Locomotor Organs, Poznan University of Medical Sciences, 28 Czerwca 1956 nr 135/147, 60-545 Poznań, Poland; awincek@ump.edu.pl (A.W.); kat.leszczynska@gmail.com (K.L.); 2 Department of Neurosurgery, Wroclaw Medical University, Borowska 213, 50-556 Wroclaw, Poland; wfortuna@onet.pl (W.F.); kchmielak@wp.pl (K.C.); p.tabakov@wp.pl (P.T.); 3 Bacteriophage Laboratory, Ludwik Hirszfeld Institute of Immunology and Experimental Therapy, Polish Academy of Sciences, Rudolfa Weigla 12, 53-114 Wrocław, Poland; 4 Neurorehabilitation Center for Treatment of Spinal Cord Injuries AKSON, ul. Bierutowska 23, 51-317 Wroclaw, Poland; sokurowski@wp.pl

**Keywords:** incomplete spinal cord injury, repetitive transcranial magnetic stimulation, cervical and thoracic spinal cord injury, rehabilitation

## Abstract

Repetitive transcranial magnetic stimulation (rTMS) may support motor function recovery in patients with incomplete spinal cord injury (iSCI). Its effectiveness mainly depends on the applied algorithm. This clinical and neurophysiological study aimed to assess the effectiveness of high-frequency rTMS in iSCI patients at the C2–Th12 levels. rTMS sessions (lasting 3–5 per month, from 2 to 11 months, 5 months on average) were applied to 26 iSCI subjects. The motor cortex was bilaterally stimulated with a frequency at 20–25 Hz and a stimulus strength that was 70–80% of the resting motor threshold (15.4–45.5% maximal output) during one therapeutic session. Surface electromyography (sEMG) recordings at rest and during maximal contractions and motor evoked potential (MEP) recordings were performed from the abductor pollicis brevis (APB) and the tibialis anterior (TA) muscles. The same neurophysiological studies were also performed in patients treated with kinesiotherapy only (K group, *n* = 25) and compared with patients treated with both kinesiotherapy and rTMS (K + rTMS). A decrease in sEMG amplitudes recorded at rest from the APB muscles (*p* = 0.001) and an increase in sEMG amplitudes during the maximal contraction of the APB (*p* = 0.001) and TA (*p* = 0.009) muscles were found in the K + rTMS group. A comparison of data from MEP studies recorded from both APB and TA muscles showed significant changes in the mean amplitudes but not in latencies, suggesting a slight improvement in the transmission of spinal efferent pathways from the motor cortex to the lower spinal centers. The application of rTMS at 20–25 Hz reduced spasticity in the upper extremity muscles, improved the recruitment of motor units in the upper and lower extremity muscles, and slightly improved the transmission of efferent neural impulses within the spinal pathways in patients with C2–Th12 iSCI. Neurophysiological recordings produced significantly better parameters in the K + rTMS group of patients after therapy. These results may support the hypothesis about the importance of rTMS therapy and possible involvement of the residual efferent pathways including propriospinal neurons in the recovery of the motor control of iSCI patients.

## 1. Introduction

An increasing number of studies provide evidence that transcranial magnetic stimulation (rTMS), together with the other classical rehabilitation methods, such as physical therapy and kinesiotherapy, may bring benefits for the recovery of sensory and motor function in patients with an incomplete spinal cord injury (iSCI) [1]. rTMS is known to evoke the long-term potentiation or depression of neuronal circuits’ activity (on the supraspinal and/or spinal levels) depending on the accepted protocol, including the widely applied stimuli algorithm [2,3,4,5,6,7]. Low-frequency rTMS (less than 20 Hz) decreases cortical excitability, whereas a higher frequency (more than 30 Hz) increases activity in the corticospinal pathway axons and reduces corticospinal inhibition. Therefore, paresis and spasticity, two main clinical symptoms in patients with an iSCI, should change following rTMS therapy [8]. Both the mechanism and structures within the central nervous system responsible for positive treatment results remain unknown. Many of the studies on the treatment of iSCI patients describe this phenomenon as “functional recovery” [9,10,11].

Our previous study on the short-term results from rTMS in patients with a C4–Th2 iSCI provided evidence of its positive effects; however, it was conducted on a limited number of participants [10]. Thus, it is crucial to ascertain the long-lasting impacts, e.g., on thoracic spinal levels, of rTMS therapy in a larger population of iSCI patients. It is also necessary to understand how rTMS therapy can be optimized in clinical practice. One of the most promising features of rTMS is the cumulative effect of several therapeutic sessions, which according to the previous descriptions appeared differently from 2 weeks to 5 months [5,7,10]. Therefore, this study aims to investigate the long-term effects of rTMS application.

Results of experimental and clinical studies indicate a group of long axonal projections of spinal neurons from cervical to lumbosacral centers, which are called propriospinal neurons (PNs). They are responsible, among other residual efferent pathways, for the coordination of locomotor mechanisms in the spinal cord and are engaged in the recovery of motor and sensory deficits in iSCI patients [1,9,12]. They are known to have primarily crossed, rather than uncrossed, projections of long descending axons in the spinal funiculi at low thoracic and upper lumbar spinal cord neuromeres, interconnecting cervical and lumbosacral centers [13]. If studies in patients with both cervical and thoracic iSCI reveal a greater improvement in motor function following rTMS and kinesiotherapy than those previously studied in patients with a C4–Th2 iSCI, the involvement of the PN system will be indicated for the recovery in spinal interconnections. Moreover, the results of the study may contribute to wider rTMS clinical applications in the treatment of iSCI patients, which was highlighted in previous studies [2,3,4,5,6,7,8].

In this study, we investigated the long-term effect of the rTMS protocol at frequencies ranging from 20 to 25 Hz and a stimulus strength that was 70–80% of the resting motor threshold in patients with C2–Th12 iSCI. The rTMS efficacy was evaluated by surface electromyography (sEMG) and motor-evoked potential (MEP) recordings. We have also compared neurophysiological data in iSCI patients treated only with kinesiotherapy and those treated with kinesiotherapy and rTMS to prove the effect of repetitive magnetic stimulation included in the current standard of care. We hypothesized that such excitation of motor cortex centers may decrease spasticity and improve motor function in iSCI patients.

## 2. Materials and Methods

The study was performed in accordance with the Declaration of Helsinki and was approved by the Bioethics Committee from the University of Medical Sciences (including studies on healthy subjects; decision no. 559/2018). Before the clinical and neurophysiological studies, 55 participants and 50 healthy volunteers declared their stable psychological and social status and signed written informed consent prior to further participation in the study.

### 2.1. Participants

**Figure 1:**
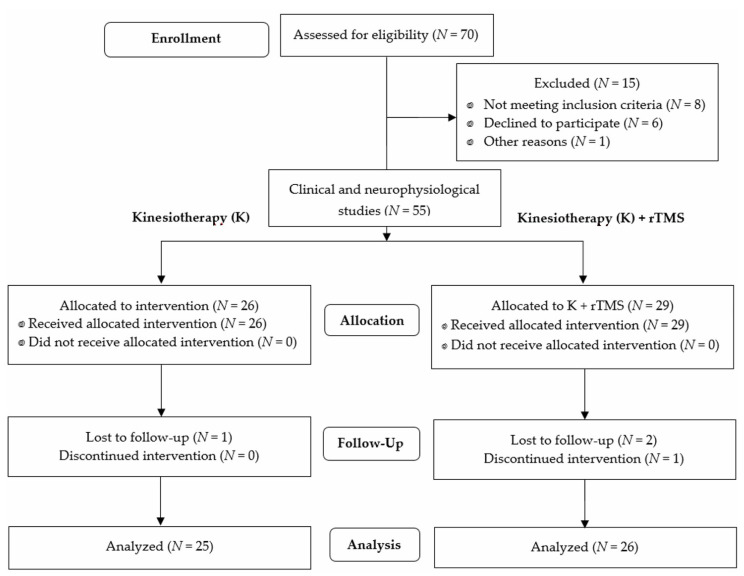
Flow chart of the study.

The preliminary sample included 70 patients with iSCI at the cervical and thoracic levels (Figure 1) that was treated surgically for spine stabilization. Fifteen were excluded because they did not meet the inclusion criteria (*n* = 8), declined to participate in the project (*n* = 6), or had died (*n* = 1). Finally, 55 patients participated in the study from February 2018 to February 2020; 26 of them with injuries at the C2–Th12 levels (C2–C7 = 15, Th1–Th12 = 11) received the kinesiotherapeutic and rTMS treatment described in Section 2.2.1 and Section 2.2.2 (K + rTMS group). The timeline between the allocation and follow-up ranged from 2 to 4 months. Eighteen men (aged between 25 and 45 years) and eight women (aged between 37 and 41 years) with similar weights and heights (59 kg and 165 cm on average, respectively) were included in this group. Another sample of 25 patients with an iSCI at cervical and thoracic levels C3–Th12 (C2–C7 = 14, Th1–Th12 = 11) was treated with kinesiotherapy only (K group, therapy described in Section 2.2.1), and was included in the study to compare the results of the applied therapies. The timelines between allocation and follow-up ranged from 1 to 4 months in the K group. This group included fourteen men (aged between 23 and 46 years) and eleven women (aged between 36 and 43 years) with similar weights and heights (62 kg and 175.3 cm on average, respectively).

Clinical studies were conducted according to the American Spinal Injury Association (ASIA) impairment scale and the tests revealed AIS C in 20 patients and D in 6 patients in the K + rTMS group and AIS C in 19 patients and D in 6 patients in the K group. Data in Table 1 summarizes the characteristics of the patients. Patients were mainly victims of car, industrial, or sports accidents. The main inclusion criteria for both groups of patients were the preservation of one-third to one-quarter of the spinal structures within fibers and neurons in the white and gray matter based on the results of MRI studies, as well as from the results of direct MEP recordings performed before treatment that confirmed the range of the iSCI. The other inclusion criteria were: first observation not less than a year since the spinal injury, agreement to participate in the project for not less than three months, no head injuries, no epilepsy episodes, no cardiovascular diseases, no psychical disorders, no pregnancy, no oncological episodes, no pacemaker or cochlear implants, no strokes nor plexopathies episodes during treatment, no inflammatory diseases nor myelopathies before the incident, written informed consent for participation in the rTMS procedures (understanding the risk), subsequent neurophysiological examinations on demand, and a stable psychological and social status. The patients who could not be treated with rTMS because of the exclusion criteria mentioned hereinabove were included in the K group.

The group of healthy volunteers (*n* = 50, 26 men and 24 women) was examined to obtain the reference values of neurophysiological recordings. The mean age of the healthy group was 38.2 ± 4.3 years (range from 26 to 45) and their heights were 155–178 cm with a mean of 168.3 ± 3.7 cm. The subjects of both patients and healthy volunteer sets did not differ significantly in age or height (*p* = 0.7). A general practitioner, neurologist, and neurosurgeon evaluated their health statuses.

### 2.2. Procedures and Intervention

#### 2.2.1. Kinesiotherapy

**Figure 2:**
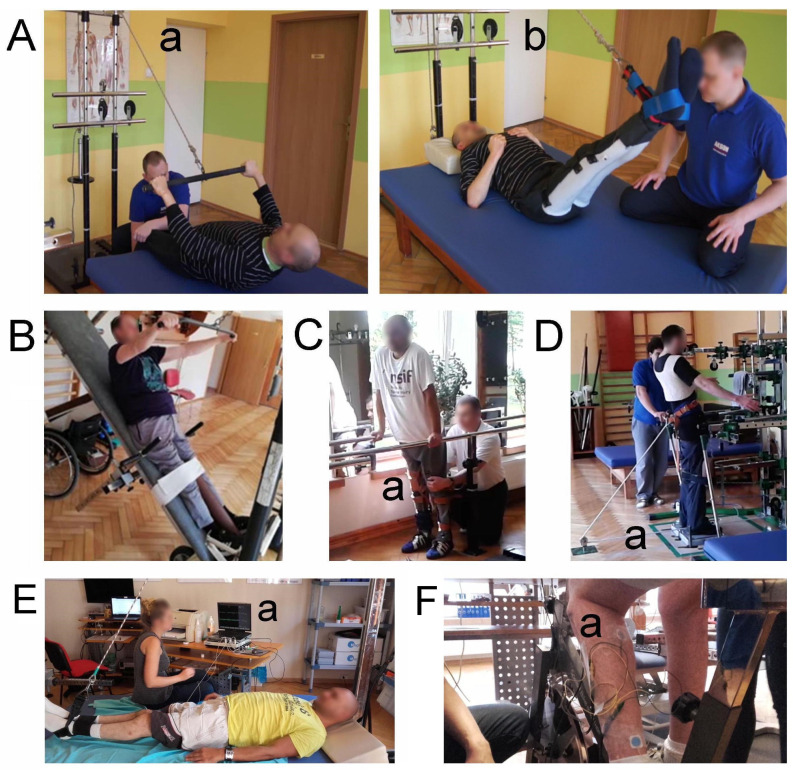
Photographs illustrating the principles of physiotherapeutic treatment and neurophysiological tests applied to the K group in this study: (**A**) a range of motion (**a**) and stretching exercises (**b**), (**B**) verticalization training, (**C**) training of locomotion on a runway with handrails with a support (**a**), (**D**) posture and balance exercises on a vibration platform (**a**), (**E**) bilateral recordings of surface electromyography (sEMG) from upper and lower extremity muscles with the visual feedback of a subject who observed the monitor with recordings (**a**), and (**F**) sEMG recordings with surface electrodes (**a**) during exercise on a stationary bicycle.

Kinesiotherapy is a part of physiotherapy; it includes the application of exercises that are supervised by a physiotherapist and are focused on improving motor and sensory function. The algorithm in this study included the same intensive programs applied in all 55 patients between the rTMS sessions, which were the only physicotherapeutic interventions. A physiotherapist carried out the programs following consultations with a neurosurgeon and neurologist in the Akson Neurorehabilitation Center in Wrocław, Poland. Sets of training were included in the rehabilitation program, as shown in Figure 2; the rehabilitation program did not differ between both study groups. One rehabilitation set lasted three months; the break between sets was longer than one month. A physiotherapist supervised patient treatment 4–5 h per day, five days a week. One session of daily training consisted of a range of motion and stretching exercises with loadings for 1 h (the magnitude of loading exercises depended on the spasticity or hypertonia level, and the minimal loading was 100 g), and was adjusted individually for certain groups of partially paralyzed muscles showing activation improvement. Depending on the spasticity or hypotonia, verticalization training was performed. The use of a verticalization bed was denied when minor neurological symptoms, such as tingling or a decrease in muscle strength or function of the autonomic nervous system, were detected. Locomotor training for 3 h on a runway with handrails with support first and later without physiotherapist assistance, as well as sensory training of posture and balance for 1 h on a specially designed vibration platform, were performed. Walking exercises were administered when the Walking Index scores increased [14]. The number of repetitions in one trial (exercise) ranged from 6 to 15, depending on the patient’s condition, five days per week.

#### 2.2.2. Repetitive Transcranial Magnetic Stimulation (rTMS)

**Figure 3:**
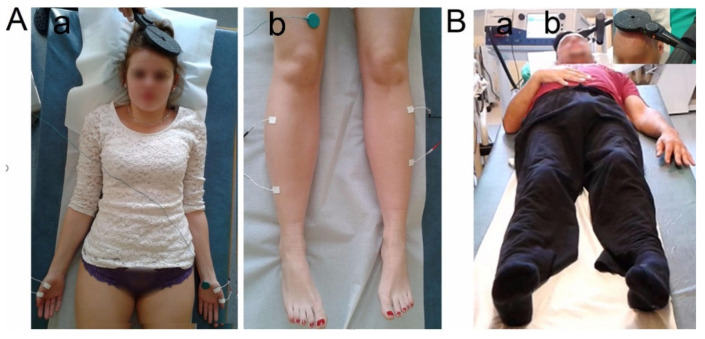
Photographs illustrating the principles of (**A**) electromyography (EMG) recorded from the upper (**a**) and lower (**b**) extremities and motor evoked potentials (MEPs) recordings and (**B**) repetitive transcranial magnetic stimulation (rTMS) treatment with a MagPro device ((**a**)—magnetic field generator; (**b**)—coil over the scalp) performed on the K + rTMS patients.

Patients received rTMS therapy in the Department of Pathophysiology of the Locomotor Organs of the Poznan University of Medical Sciences, Poland, and the Neurorehabilitation Center for Treatment of Spinal Cord Injuries AKSON in Wroclaw, Poland. Both facilities applied the same rTMS algorithm. A maximum of 15 sessions of rTMS was applied within five months on average (±1 month). The MagPro R30 and MagPro X100 magnetic stimulator with MagOption Medtronic (Medtronic A/S, Skovlunde, Denmark) were used for the rTMS therapy and for performing the motor evoked potentials (MEPs) diagnostic studies (Figure 3). Treatment was induced with a circular coil (C-100, 12 cm in diameter) that was placed over the scalp in the area of the M1 motor cortex, targeted with an angle for the corona radiata excitation, where the fibers of the corticospinal tract for the upper and lower extremities originated (Figure 3B(b)). A train of stimuli with a maximum limit of 2–4 tesla (T) on the surface of a patient’s head was induced from the magnetic field generator (Figure 3B(a)). The magnetic field stream delivered from the coil had a strength that was 70–80% of the resting motor threshold (RMT; 0.84–0.96 T); this field excited all neural structures up to 3–5 cm deep. It is possible that the cells of origin of the rubrospinal tract in the midbrain were also excited [1,10]. The RMT test performed as a single stimulus that was 50% of the maximal stimulus output (MSO) was used before and at the end of each course. Afterward, the individual RMT, as the minimum magnetic stimulus intensity required to elicit an MEP > 50 μV peak-to-peak amplitude above the background electromyographic activity in the relaxed key muscles, was determined. sEMG was performed on the abductor pollicis brevis (APB) and tibialis anterior (TA) muscles (Figure 3A). A single stimulus with a lower intensity (usually 38–40% of the MSO) was used before the rTMS therapeutic sessions. It evoked minimal muscle twitch for both the APB and TA. The maximal stimulus intensity that was used for diagnostic purposes was not more than 70–80% of the RMT. Diagnostic stimulations had the same locations bilaterally over the scalp as they did during the therapies. The stimuli had individually designed algorithms based on repetitive sets of neurophysiological tests and the patient’s current clinical state at a subsequent stage of observation. Although the main schedule was kept the same for all patients, the frequency of the applied stimuli was adjusted higher (from 15 up to 25 Hz), depending on the severity of the spinal injury (worse MEP results) and neurogenic dysfunction detected in muscles (low-frequency and low-amplitude sEMG recordings). For example, when the amplitudes of the MEP and sEMG (during the attempt of maximal contraction) recordings were about 100 μV and the frequency index was 1, the algorithm required the application of higher frequency rTMS trains up to 25 Hz. If results were observed to be better (an increase in the amplitude and the frequency index was recorded), the frequency of rTMS was decreased toward 15 Hz. One session of therapy consisted of 3–5 rTMS sessions in a month, and one session per day was conducted; the motor cortex’s bilateral stimulation was performed for about 10 min with 10 min intervals. Patients whose values in the MEP and sEMG recordings improved in the second observation, and did not report the symptoms of muscle fatigue or general tiredness, received a greater number of rTMS sessions. In general, patients received 1600 biphasic pulses, 800 pulses for each hemisphere during each session. The parameters were as follows: 15–25 Hz—frequency of the applied stimuli, 2 s trains of 40 pulses, 28 s—the interval between trains of stimuli, and a strength of about 70–80% of the RMT. The whole observation period of rTMS application was from 2 to 11 months, 5 months on average. None of the patients reported the rTMS as being painful, though they felt a slight spread of current to the upper and lower extremities; they were always awake and cooperating. None of the patients received antispastic drugs when the rTMS therapy was applied. The neurologist advised not to take antispastic drugs after the statement of participation was signed up at the beginning of the project.

### 2.3. Neurophysiological Studies

Patients were examined using neurophysiological methods in the Department of Pathophysiology of the Locomotor Organs of the Poznan University of Medical Sciences, Poland, except for the set of clinical studies included in the ASIA scale evaluation. Studies of efferent spinal transmission and muscle motor unit activity were performed in three periods of observations, before and after each rTMS session in K + rTMS group and separately in the K group of patients. The KeyPoint Diagnostic System (Medtronic A/S, Skovlunde, Denmark) was used for MEP and sEMG recordings in the iSCI patients and healthy volunteers (*n* = 50, control group) to obtain the reference values of neurophysiological recordings for comparison (Table 2).

#### 2.3.1. Surface Electromyography Recordings (sEMG)

**Figure 4:**
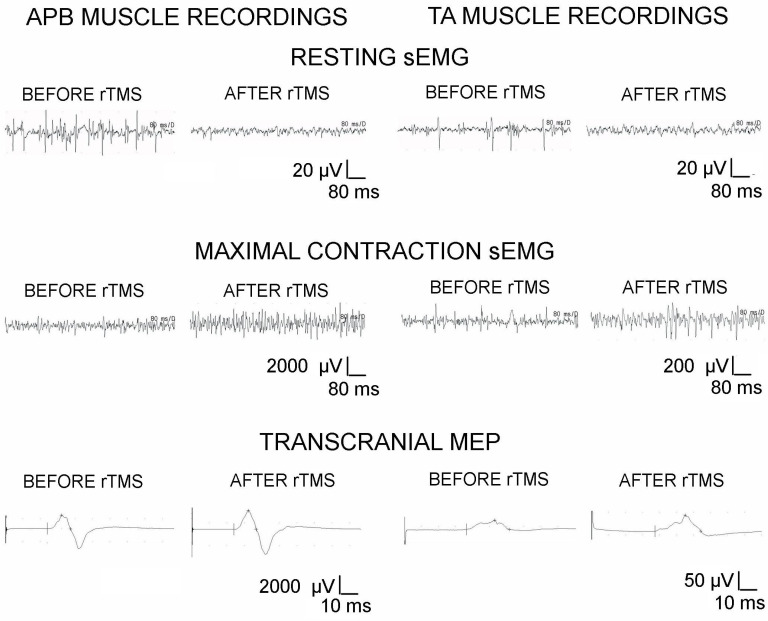
Examples of sEMG and MEP recordings from one iSCI patient before the first rTMS session and after the last therapeutic session. Calibration bars for different amplifications and time bases are presented.

sEMG activity was recorded from the bilateral abductor pollicis brevis (APB) and tibialis anterior (TA) muscles with surface electrodes that measured the amplitudes and frequencies at rest and during maximal contraction attempts lasting 5 s. Standard disposable Ag/AgCl recording surface electrodes that had an active surface of 5 mm^2^ were used. sEMG recordings were performed with an active electrode placed on the muscle belly, while the referencing electrode was placed on its distal tendon, according to the Guidelines of the European Federation of Clinical Neurophysiology. The ground electrode was located on the distal part of the leg. As is commonly done, the upper 10 kHz and lower 20 Hz filters of the recorder were set. Recordings were made at the time base of 80 ms/D and an amplification of 20–1000 µV. The amplitude of the sEMG recordings below 20–25 µV at rest identified proper muscle tension [15]. The average amplitude parameters (minimum–maximum, i.e., the peak-to-peak of recruiting motor unit action potential deflection with reference to the isoelectric line measured in µV), and motor unit firing frequencies (the number of recruited motor unit action potentials (in Hz)) were analyzed in recording during maximal contraction. The amplitudes below 900 and 600 µV suggested the pathological recruitment of motor unit activity during the maximal contraction of APB and TA muscles, respectively (Figure 4). In the sEMG studies, the neurophysiologist verbally encouraged the patients with iSCI to make three attempts of maximal contraction. Participants were instructed to contract the tested muscle as hard and as fast as possible until the neurophysiologist requested them to finish the attempt. The recruitment of the muscle motor units heard by the patients in the loudspeaker of the recorder motivated the subjects in a biofeedback way. The test was conducted three times, with a 1 min resting period between each set of muscle contractions; the recording with the highest amplitude (in µV) and frequency (in Hz) parameters was selected for analysis. In some patients with worse sEMG results in a supine position due to muscle fatigue, dynamic exercises (tests) on stationary bicycles were applied to increase their motivation during simultaneous sEMG recordings. The frequency index from 3 to 0 (3 = 95–70 Hz—normal; 2 = 65–40 Hz—moderate abnormality; 1 = 35–10 Hz—severe abnormality; 0 = no contraction) was used according to the description elsewhere [11,15,16], with the use of automatic analyzing software included in the KeyPoint System, which were compared to the online readings of sEMG recordings.

#### 2.3.2. Motor Evoked Potentials (MEPs) Recordings 

Motor evoked potentials were elicited via a transcranial magnetic single stimulus using the same magnetic coil as for rTMS purposes and recorded with surface electrodes from the APB and TA muscles. The latency and amplitude parameters were analyzed as the primary outcome measures for assessing the primary motor cortex’s output and evaluating the efferent transmission of neural impulses to effectors via spinal cord descending tracts. Consecutive tracking attempts were made to find the optimal stimulation location (a hot spot in the area where the rTMS elicited the largest MEP amplitude), distanced 5 mm each other. The amplitudes below 1125 μV and 1200 μV for the MEPs recorded from APB and TA muscles in response to an applied single magnetic pulse indicated pathological transmission in descending spinal cord pathways of the axonal type, respectively. The methodology of MEP recordings was described in detail elsewhere [10,11].

### 2.4. Data Analysis and Statistics

Data were analyzed with Statistica, version 13.1 (StatSoft, Kraków, Poland). Descriptive statistics were reported as minimal and maximal values (range), with mean or median and standard deviation (SD). The normality distributions were studied with Shapiro–Wilk tests and the homogeneity of variances were studied with Levene’s tests. Frequency index data were of the ordinal scale type, while amplitudes and latencies were of the interval scale type. However, they did not represent a normal distribution; therefore, the non-parametric tests had to be used. None of the collected data represented a normal distribution or were of the ordinal scale type (frequency index data); therefore, the Wilcoxon’s signed-rank tests were conducted to compare the differences between results obtained before and after treatment, as well as to compare results at the beginning of the treatment using rTMS (first observation—before treatment) and at the end (third observation—after treatment). Any *p*-values ≤ 0.05 were considered statistically significant. The statistical software was used to determine the required sample size using the primary outcome variable of the sEMG amplitude recorded from the APB and TA muscles at rest before and after treatment with a power of 80% and a significance level of 0.05 (two-tailed). The mean and standard deviation (SD) were calculated using the data from the first seven patients, and the sample size software estimated that more than 21 patients were needed for the purposes of this study.

## 3. Results

Figure 4 presents examples of the sEMG and MEPs recorded in one patient with iSCI at Th11–Th12. The rTMS therapy evoked a decrease in sEMG amplitudes in both the APB and TA muscles at rest and a greater increase in amplitude than the frequency in the sEMG recordings from the APB muscles than the TA muscles. The above phenomena can be interpreted as a decrease in spasticity and an improvement in muscle motor unit activity in this patient. Similar changes induced by the rTMS were statistically shown to be significant in the entire patient population when comparing results recorded before and after therapy (Table 3).

**Figure 5:**
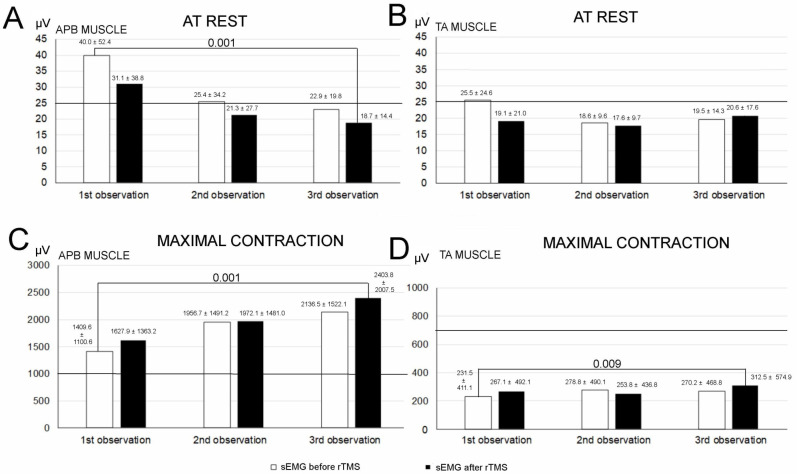
Comparison of mean amplitudes (expressed in µV) of the sEMG performed on the APB (**A**,**C**) and TA (**B**,**D**) muscles at rest (**A**,**B**) and during the attempt of maximal contraction (**C**,**D**) before and after each therapeutic session. Horizontal lines refer to mean values recorded in healthy subjects (control group).

The comparison of the data from the first and last observations shown in Figure 5A provides evidence for a significant decrease in the mean sEMG amplitude parameter recorded at rest from ABP muscles (at *p* = 0.001) in the whole population of iSCI patients following the applied therapy. However, such a phenomenon of reaching the lower physiological limit of no more than 20 µV was also observed in the sEMG recordings at rest in the TA muscles (Figure 5B).

The studied group of iSCI patients did not present the pathology of APB muscle motor units during maximal contractions before treatment, but the data in Figure 5C that indicates the improvement in their activity after the rTMS therapy, which was significant at *p* = 0.001, is convincing (sEMG amplitude parameter more than 1000 µV). All the iSCI patients showed TA muscle activity below the lower physiological limit (average value of amplitude = 725 µV), and rTMS therapy provided a slight but statistically significant (*p* = 0.009) increase in the sEMG amplitude parameter recorded during a maximal contraction (Figure 5D). 

A comparison of the data from MEP studies recorded from both APB and TA muscles presented in Table 3 shows significant changes in the mean amplitudes but not in latencies, suggesting a slight improvement in the transmission of spinal efferent pathways from the motor cortex to the lower spinal centers. However, it should be remembered that abnormalities were not recorded before therapy in the MEP from the upper extremity muscles; those from lower extremity muscles were only residually recorded (Figure 4).

Data in Table 4 indicate the highest percentage of improvement in parameters of the sEMG (both at rest and during maximal contraction) and MEP recordings in the K + rTMS group than in the K group. This refers not only to recordings performed from muscles of the upper but also lower extremities. The neurophysiological parameters recorded in both groups of iSCI patients did not differ significantly before the therapy. However, they were significantly different in the final observation, with *p*-values ranging from 0.04 to 0.05.

## 4. Discussion

rTMS is a non-invasive technique for neuromodulation and has therapeutic potential for motor rehabilitation following an iSCI [2,3,4,5,6,7,8]. In our previous study, patients who suffered a C4–Th2 iSCI that were treated with 20–22 Hz rTMS showed an improvement of functional status by means of spasticity decrease and a greater increase in muscle motor unit activity in upper than in lower extremities [10]. A statistically significant improvement in the transmission of efferent neural impulses within spinal pathways to cervical centers and upper extremity muscles was observed in the MEP recordings. In this study, we chose patients with similar injuries at the cervical or thoracic spinal levels, assumed from MRI coronal and vertical planes, where one-third to one-quarter of the neural structures were preserved, as well as from the results of direct MEP recordings performed before treatment, which confirmed the range of each iSCI. Both populations of C4–Th2 and C2–Th12 patients were different regarding the duration of treatment: up to 5 months and from 2 to 11 months (5 months of average), respectively. The results of the comparative neurophysiological tests in this study on patients with C2–Th12 iSCI provide evidence of the similarity in the above-mentioned phenomena found in the C4–Th2 iSCI patients, with additional improvements in muscle motor unit activity in the lower extremities. The results of this study also provide evidence regarding the improvement of the efferent transmission of neuronal impulses to the spinal motor centers in neuromeres below the level of the injury, as shown by the better results of the MEPs recordings from the TA muscles than in the study of Leszczyńska et al. [10]. Throughout the whole population of patients, a significant improvement of the sEMG parameters recorded from the APB muscle was commonly observed in the second period of observations (see Figure 5). It is difficult to ascertain the exact time point of the applied rTMS effectiveness, but it seems to appear after 50% of the scheduled rTMS sessions in the majority of patients. Significant changes in amplitudes parameters of MEP recordings following rTMS suggest that the maintenance of the efferent spinal cord’s transmission was at least at the same level as it is before therapy. If an amplitude of MEP recordings from lower extremity muscles reflects the number of axons in the white matter actively transmitting neuronal impulses, it may also indicate the therapy’s protective mechanism was inhibiting degenerative changes of the spinal structures. Patients of both groups were treated with the same algorithms of physical therapy and kinesiotherapy, which implied additional factors influencing the electrophysiological parameters of the C2–Th12 iSCI patients.

The data in Table 4 showing results of neurophysiological recordings in the iSCI patients of both groups clearly provides evidence of the superiority of treatment with kinesiotherapy and rTMS (K + rTMS group) in comparison with the effects found in patients that were treated with only kinesiotherapy (K group). This part of the study appears to be necessary to confirm the importance of rTMS therapy in future clinical practice.

The transmission in the corticospinal pathway is crucial for the recovery of motor function in iSCI patients. Functional recovery is possible either due to the sprouting of new axons, the transmission of neuronal impulses via rudimentary saved corticospinal axons, or the system of intersegmental crossed projections of propriospinal fibers [1,9]. The propriospinal neurons with crossed thoracic and upper lumbar axonal projections, which were partially preserved in patients under this study, among other residual efferent pathways, could modify the functional recovery by transmitting efferent impulses from the cervical to lumbosacral levels. Their cells of origin at the cervical levels are known to be modulated by inputs from supraspinal centers, e.g., corticospinal and rubrospinal systems, which were excited with magnetic field trains of impulses during therapeutic sessions [17,18]. However, it cannot be ignored that some spared fibers in lateral funiculi belonging to these systems participate in neural transmission as well. On the other hand, propriospinal neurons are known to especially compensate for the function of injured spinal cord pathways in long-term experimental and clinical observation [12]. They play an essential role in motor control (including locomotion) and sensory processing. They are an important substrate for spinal cord “bridging” in incomplete lesions and contribute to the plastic reorganization of spinal circuits [1]. We believe that our study results prove the effectiveness of rTMS’s ability to elicit one of the systems mentioned above. Previous studies have demonstrated that PNs might also be an essential substrate for recovery in iSCI patients, as they contribute to “functional recovery” [19,20,21]. Considering the contemporary results of studies on patients with both cervical and thoracic iSCIs, which revealed a better improvement of motor function following rTMS and kinesiotherapy compared with those previously studied in patients with a C4–Th2 iSCI, involvement of the PN system for recovery in spinal interconnections is indicated. We assume that in our iSCI patients, PNs compensated for abnormalities in the transmission of neural impulses in white matter fibers and improved the activity of synaptic connections at the motoneuronal level. The thoracic levels of injury play a significant role as a structural basis of electrophysiological improvements since axons of propriospinal neurons cross at this level and may be the additional way of motor transmission compensation to the residual fibers after iSCI.

This study used a modified rTMS algorithm of stimulation that was described by other authors to treat patients with iSCI [2,3,4,5,6,7,8], which evoked positive effects toward improving motor function and reducing spasticity. This may imply that 20–25 Hz rTMS that was adjusted according to each patient’s needs based on consecutive neurophysiological test results enhanced the corticospinal synaptic transmission, and this effect was sustained for at least five months. Only a few of the previous studies utilized repetitive testing of sEMG [6,7] and MEP recordings [3,8,11] in the evaluation of positive treatment results in patients with an iSCI. Most of the previous works used clinical methods of functional assessments of patients with iSCI, such as gait improvement analysis; therefore, the presented study results cannot be directly compared. 

### Limitations

The heterogeneity of the injuries in the patients with an iSCI in this study was its main limitation, although we attempted to recruit subjects with rigorous enrollment rules, such as the ASIA scale evaluation. By contrast, the number of presented patients and performing three trials in long-lasting observations compared to previous results seem to be the main advantages, as well as the comparison of results obtained in the group treated with kinesiotherapy with the group of patients treated with both kinesiotherapy and rTMS. We are aware that having a non-treated identical control group (ideally submitted to a placebo-like intervention) in our study would broaden the scope of the functional regeneration mechanism from the point of basic neuroscience. On the other hand, it would be difficult for iSCI patients looking for any improvement in their health status following rTMS to understand the importance of a placebo approach in the project, as they need to be informed in advance. The other limitation is the uncertainty that muscle fiber atrophy in iSCI patients might be partially caused by superimposing chronic immobility, which makes positive results of any treatment applied to iSCI patients difficult to interpret.

## 5. Conclusions

The comparison of the results of this study regarding the influence of rTMS at 20–25 Hz in patients with C2–Th12 iSCI in a long-term observation with previous data regarding the outcomes of similar treatment in C4–Th2 iSCI patients may confirm the hypothesis about the significance of the propriospinal system and other residual efferent pathways in the recovery of motor control. The proposed rTMS algorithm reduced spasticity symptoms more in the upper extremity muscles and improved the recruitment of upper and lower extremity muscle motor units and MEPs parameters, which may imply a slight improvement in the transmission of efferent neural impulses within spinal pathways. The comparison of neurophysiological data in iSCI patients treated only with kinesiotherapy (K group) and those treated with kinesiotherapy and rTMS (K + rTMS group) may indicate the importance of including the rTMS therapy in the current standard of care. rTMS was confirmed as a novel, non-invasive, and safe therapeutic method for enhancing voluntary motor output in motor disorders affecting the descending spinal pathways. Its potential clinical application seems to be more effective in conjunction with kinesiotherapeutic treatment, which requires further extensive studies.

## Figures and Tables

**Table 1:** Characteristics of the subjects studied in two groups before treatment.

	K Group		K + rTMS Group	
	Mean ± SD	Min–Max	Mean ± SD	Min–Max
Age	36.7 ± 5.3	23–46	37.3 ± 4.7	25–45
Height (cm)	175.3 ± 4.3	165–181	165.0 ± 4.3	158–172
Weight (kg)	62.0 ± 6.1	51–79	59.0 ± 5.5	51–83
	*n*		*n*	
AIS scale	C = 19 D = 6		C = 20 D = 6	
Spinal injury level	C2–C7 = 14 Th1–Th12 = 11		C2–C7 = 15 Th2–Th12 = 11	

K group—iSCI patients treated with kinesiotherapy, K + rTMS—iSCI patients treated with kinesiotherapy and repetitive transcranial magnetic stimulation.

**Table 2:** Reference values of surface electromyography (sEMG) parameters that were recorded at rest and during an attempt of maximal contraction, and MEP parameters that were recorded following transcranial magnetic stimulation in a group of healthy volunteers (*n* = 50). Ranges and mean or median values are presented.

Recording	Measured Parameter	Healthy Volunteers
sEMG APB	Amplitude at rest (µV)	15–30 25.3 ± 2.6
	Amplitude during maximal contraction (µV)	900–1800 1025 ± 105
	Frequency index	3-3 3.0
sEMG TA	Amplitude at rest (µV)	15–30 25.6 ± 2.2
	Amplitude during maximal contraction (µV)	600–1450 725 ± 110
	Frequency index	3-3 3.0
MEP APB	Amplitude (µV)	1125–3650 1662.5 ± 472.8
	Latency (ms)	18.6–22.7 20.65 ± 2.05
MEP TA	Amplitude (µV)	1200–2975 1656 ± 370.7
	Latency (ms)	25.9–31.75 28.8 ± 1.7

Modified frequency index (3-0)—frequency of motor unit action potentials recruitment during maximal contraction sEMG recording: (3 = 95–70 Hz—normal; 2 = 65–40 Hz—moderate abnormality; 1 = 35–10 Hz—severe abnormality; 0 = no contraction); sEMG—surface electromyography; MEP—motor evoked potential; APB—abductor pollicis brevis muscle; TA—tibialis anterior muscle.

**Table 3:** Comparison of results from neurophysiological studies in patients (*n* = 26) at certain periods of observation. Ranges, mean or median values, and standard deviations are presented.

Recording	Measured Parameter	1st Observation (Before Treatment)			2nd Observation (After 2–3 Months of Treatment)			3rd Observation (After 5 Months of Treatment)			Difference 1st vs. 3rd
		Before rTMS Sessions	After rTMS Sessions	*p*	Before rTMS Sessions	After rTMS Sessions	*p*	Before rTMS Sessions	After rTMS Sessions	*p*	*p*
sEMG APB	Amplitude at rest (µV)	5–200 40.0 ± 52.4	5–200 31.1 ± 38.8	0.011 *	5–200 25.4 ± 34.2	5–200 21.3 ± 27.7	0.007 *	10–100 22.9 ± 19.8	10–100 18.7 ± 14.4	0.004 *	<0.001 *
	Amplitude during maximal contraction (µV)	100–4000 1409.6 ± 1100.6	100–7000 1627.9 ± 1363.2	0.002 *	100–6000 1956.7 ± 1491.2	100–7000 1972.1 ± 1481.0	0.334	100–6000 2136.5 ± 1522.1	100–10500 2403.8 ± 2007.5	0.030 *	<0.001 *
	Frequency index	1-3 2.2 ± 0.7	1-3 2.2 ± 0.7	0.779	1-3 2.1 ± 0.7	1-3 2.2 ± 0.7	0.128	1-3 2.5 ± 0.7	0-3 2.5 ± 0.7	0.767	0.003 *
sEMG TA	Amplitude at rest (µV)	5–200 25.5 ± 34.6	5–150 19.1 ± 21.0	0.021 *	10–50 18.6 ± 9.6	10–50 17.6 ± 9.7	0.046 *	10–100 19.5 ± 14.3	10–100 20.6 ± 17.6	0.917	0.331
	Amplitude during maximal contraction (µV)	0–2000 231.5 ± 411.1	0–2200 267.1 ± 492.1	0.064	0–2000 278.8 ± 490.1	0–1700 253.8 ± 436.8	0.069	0–2100 270.2 ± 468.8	0–3000 312.5 ± 574.9	0.05 *	0.009 *
	Frequency index	0-3 0.9 ± 1.0	0-3 0.9 ± 0.9	1.0	0-3 1 ± 1.0	0-3 1 ± 1.0	1.0	0-3 0.9 ± 1.0	0-3 1.0 ± 1.1	0.109	0.320
MEP APB	Amplitude (µV)	50–12500 2052.9 ± 2511.0	0–10000 1960.6 ± 2294.8	1.0	0–10000 2351.9 ± 2599.3	0–12000 2353.8 ± 2911.2	0.602	100–15000 1942.3 ± 2668.9	100–11000 2725.1 ± 2524.1	0.05 *	0.008 *
	Latency (ms)	16.8–43.5 23.4 ± 5.2	0–37.7 22.7 ± 5.4	0.373	0–38.7 22.3 ± 5.4	0–38.7 22.7 ± 5.3	0.172	15.5–33.4 22.3 ± 3.9	14.6–33.0 23.3 ± 4.5	0.655	0.081
MEP TA	Amplitude (µV)	0–1000 76.9 ± 162.8	0–1000 80.5 ± 169.4	0.530	0–2200 128.8 ± 354.7	0–1100 107.9 ± 250.7	0.683	0–800 78.8 ± 157.6	0–1100 162.1 ± 123.6	0.05 *	0.05 *
	Latency (ms)	0–49.3 29.1 ± 18.5	0–48.0 28.7 ± 18.5	0.691	0–45.1 28.5 ± 16.4	0–45.1 28.3 ± 16.6	0.687	0–45.0 28.5 ± 17.6	0–45.0 28.1 ± 15.6	0.052	0.332

* p < 0.05; modified frequency index (3-0)—frequency of motor units action potentials recruitment during maximal contraction sEMG recording: (3 = 95–70 Hz—normal; 2 = 65–40 Hz—moderate abnormality; 1 = 35–10 Hz—severe abnormality; 0 = no contraction); rTMS—repetitive transcranial magnetic stimulation; sEMG—surface electromyography; MEP—motor evoked potential; APB—abductor pollicis brevis muscle; TA—tibialis anterior muscle. * Significant *p*-values are marked bold.

**Table 4:** Comparison of results from neurophysiological studies in patients from the K group (treated with kinesiotherapy only) and the K + rTMS group (treated with kinesiotherapy and repetitive transcranial magnetic stimulation). Ranges, mean or median values, and standard deviations are presented.

Recording	Measured Parameter	K Group			K + rTMS Group			K vs. K + rTMS Difference before	K vs. K + rTMS Difference after
		Before	After	Change (%)	Before	After	Change (%)	*p*	*p*
sEMG APB	Amplitude at rest (µV)	5–150 45.3 ± 22.1	5–200 35.7 ± 24.3	21.1	5–200 40.0 ± 52.4	10–100 18.7 ± 14.4	53.2	0.06	0.03 *
	Amplitude during maximal contraction (µV)	100–4500 1443.7 ± 1241.7	100–6500 1445.1 ± 1126.8	0.09	100–4000 1409.6 ± 1100.6	100–10500 2403.8 ± 2007.5	41.3	0.13	0.03 *
	Frequency index	1–3 2.0 ± 0.5	1–3 2.0 ± 0.6	0	1–3 2.2 ± 0.7	0–3 2.5 ± 0.7	12.0	0.06	0.04 *
sEMG TA	Amplitude at rest (µV)	10–100 22.1.6 ± 6.4	10–50 22.3 ± 7.1	0.89	5–200 25.5 ± 34.6	10–100 20.6 ± 17.6	19.2	0.07	0.06
	Amplitude during maximal contraction (µV)	0–1900 245.8 ± 381.2	0–2700 246.5 ± 321.3	0.28	0–2000 231.5 ± 411.1	0–3000 312.5 ± 574.9	25.9	0.08	0.05 *
	Frequency index	0–3 1 ± 1.0	0–3 1 ± 1.0	0	0–3 0.9 ± 1.0	0–3 1.0 ± 1.1	10.0	0.06	0.06
MEP APB	Amplitude (µV)	50–11200 1925.3 ± 1724.3	50–1050 2105.6 ± 1855.3	9.5	50–12500 2052.9 ± 2511.0	100–11000 2725.1 ± 2524.1	24.6	0.07	0.05 *
	Latency (ms)	16.2–33.4 23.1 ± 4.6	17.1–34.6 23.6 ± 3.9	2.1	16.8–43.5 23.4 ± 5.2	14.6–33.0 23.3 ± 4.5	0.42	0.14	0.13
MEP TA	Amplitude (µV)	0–1200 77.1 ± 133.5	0–1000 100.9 ± 131.6	23.5	0–1000 76.9 ± 162.8	0–1100 162.1 ± 123.6	52.5	0.11	0.05 *
	Latency (ms)	0–50.1 30.4 ± 17.1	0–49.2 29.4 ± 13.6	3.2	0–49.3 29.1 ± 18.5	0–45.0 28.1 ± 15.6	3.4	0.13	0.12

K group—kinesiotherapy treated group of patients *n* = 25, K + rTMS group—kinesiotherapy and rTMS treated group of patients *n* = 26. * Significant *p*-values are marked bold.

## Data Availability

All the data generated or analyzed during this study are included in this published article.
